# Hepatic excretory function in sepsis: implications from biophotonic analysis of transcellular xenobiotic transport in a rodent model

**DOI:** 10.1186/cc12606

**Published:** 2013-04-10

**Authors:** Falk A Gonnert, Peter Recknagel, Ingrid Hilger, Ralf A Claus, Michael Bauer, Andreas Kortgen

**Affiliations:** 1 Integrated Research and Treatment Center - Center for Sepsis Control and Care, Jena University Hospital, Erlanger Allee 101, 07747 Jena, Germany; 2 Department of Anesthesiology and Intensive Care Therapy, Jena University Hospital, Erlanger Allee 101, 07747 Jena, Germany; 3 Institute of Diagnostic and Interventional Radiology, Forschungszentrum Lobeda, Jena University Hospital, Erlanger Allee 101, 07747 Jena, Germany

## Abstract

**Introduction:**

Hepatobiliary elimination of endo- and xenobiotics is affected by different variables including hepatic perfusion, hepatocellular energy state and functional integrity of transporter proteins, all of which are altered during sepsis. A particular impairment of hepatocellular transport at the canalicular pole resulting in an accumulation of potentially hepatotoxic compounds would have major implications for critical care pharmacology and diagnostics.

**Methods:**

Hepatic transcellular transport, that is, uptake and hepatobiliary excretion, was studied in a rodent model of severe polymicrobial sepsis by two different biophotonic techniques to obtain insights into the handling of potentially toxic endo- and xenobiotics in sepsis. Direct and indirect *in vivo *imaging of the liver was performed by intravital multifluorescence microscopy and non-invasive whole-body near-infrared (NIRF) imaging after administration of two different, primarily hepatobiliary excreted xenobiotics, the organic anionic dyes indocyanine green (ICG) and DY635. Subsequent quantitative data analysis enabled assessment of hepatic uptake and fate of these model substrates under conditions of sepsis.

**Results:**

Fifteen hours after sepsis induction, animals displayed clinical and laboratory signs of multiple organ dysfunction, including moderate liver injury, cholestasis and an impairment of sinusoidal perfusion. With respect to hepatocellular transport of both dyes, excretion into bile was significantly delayed for both dyes and resulted in net accumulation of potentially cytotoxic xenobiotics in the liver parenchyma (for example, specific dye fluorescence in liver at 30 minutes in sham versus sepsis: ICG: 75% versus 89%; DY635 20% versus 40% of maximum fluorescence; *P *< 0.05). Transcutaneous assessment of ICG fluorescence by whole body NIRF imaging revealed a significant increase of ICG fluorescence from the 30th minute on in the bowel region of the abdomen in sham but not in septic animals, confirming a sepsis-associated failure of canalicular excretion.

**Conclusions:**

Hepatocytes accumulate organic anions under conditions of sepsis-associated organ dysfunction. These results have potential implications for monitoring liver function, critical care pharmacology and the understanding of drug-induced liver injury in the critically ill.

## Introduction

Sepsis reflects a systemic inflammatory syndrome in response to an infection and represents the leading cause of death in the intensive care unit [1]. A hallmark of this disease is (multiple) organ dysfunction, including liver dysfunction, ultimately determining the outcome of sepsis.

In critically ill patients, liver dysfunction can vary from subclinical injury to overt failure. An ever growing body of evidence supports the concept that among the different functions of the liver primarily the hepatic excretory function, that is, the elimination of endo- and xenobiotics, is impaired during the course of sepsis resulting in cholestasis [2-6]. Sepsis-associated liver dysfunction is traditionally viewed as a late feature of critical illness indicated by jaundice and hyperbilirubinemia. However, recent studies have revealed liver dysfunction as an early event in sepsis [4,7].

Hepatic excretory function is mediated by various factors including hepatic perfusion, binding to plasma proteins, hepatocellular energy state, uptake across the basolateral membrane, intracellular binding to proteins with subsequent transcellular transport, metabolism and canalicular excretion into bile [8]. In sepsis, each individual step is likely to be influenced by cytokines and other inflammatory mediators [9].

**Figure 1:**
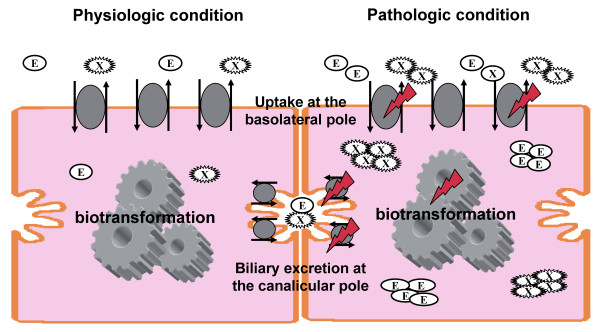
**Simplified illustration of sepsis-associated impairment of hepatobiliary excretion**. Endo- and xenobiotics are taken up from the bloodstream via transport proteins at the basolateral pole. After transcellular transport with or without biotransformation, endo- and xenobiotics are biliary excreted via transport proteins at the canalicular pole. Sufficient hepatic excretory function depends on various factors which all can be impaired under septic conditions. Atorvastatin can serve as a typical example for profoundly impaired pharmacokinetics under septic conditions leading to supratherapeutic plasma levels after a single oral dose [23]. E, endobiotics; X, xenobiotics).

Of note, recent reports hint at a differential susceptibility of the two polar surfaces of hepatocytes with the canalicular pole being more sensitive to endotoxin and cytokines [3,4,7,10]. Maximal basolateral uptake rate exceeds canalicular efflux due to a much larger surface area on the sinusoidal side of the cell. Given the particular susceptibility of canalicular transporters to endotoxin and cytokines, impaired biliary excretion may lead to intracellular accumulation of potentially hepatotoxic compounds in sepsis [11] (Figure 1). This is particularly pertinent for critically ill patients due to the large amount of drugs they receive.

Biophotonics, that is, the development and application of light-based technologies, particularly imaging techniques, to study biological processes is a rapidly evolving scientific field in the life sciences and medicine. We have previously reported a technique for investigation of hepatobiliary excretory function in the rat by intravital multifluorescence microscopy enabling an assessment of not only uptake, but also transcellular transport and biliary excretion of two different model substrates [12]. This approach was established with indocyanine green (ICG), a well characterized, near-infrared FDA-approved fluorescent dye that is widely used in medical diagnostics including quantitative liver function testing. To optimize visualization, the hemicyanine dye DY635 was validated for assessing hepatic excretory function.

In the present study, we applied this technique in a fluid-resuscitated rat model of polymicrobial sepsis. In addition, we report a non-invasive biophotonic approach capable of specifically monitoring the canalicular pole by whole body near-infrared fluorescence (NIRF) imaging. These approaches allowed us to analyse sepsis-associated impairment of hepatic excretory function, demonstrating potential implications for the clinical setting.

## Materials and methods

### Animals

Male Wistar rats (12 to 16 weeks old, body weight 400 ± 50 g; Harlan Laboratories, Horst, Netherlands) were used in this study. All animal procedures were approved by the regional animal welfare committee (Thuringian State Office for Food Safety and Consumer Protection, Department for Animal Welfare, Bad Langensalza, Thuringia, Germany). Body temperature was maintained at 37°C during all experimental procedures. All surgical preparations were performed under aseptic conditions. Animals were instrumented after application of local anesthetics under desflurane anesthesia (Baxter, Munich, Germany).

### Sepsis model

A reliable and reproducible sepsis model reflecting many features of human sepsis was applied [13,14]. In brief, this model relies on polymicrobial peritoneal infection induced by intraperitoneal injection of a standardized, microbiologically characterized stool suspension resulting in severe peritoneal and systemic inflammation with consecutive multiple organ dysfunction and finally death of all animals after approximately two days if no antibiotics are administered. Fifteen hours post insult the expected mortality varies between 15% and 30%.

Animals were randomly allocated to the sham or septic group (septic group, 1.75 ml/kg stool suspension, diluted (1:4) in saline; sham group, a similar volume of saline). Both groups received fluid resuscitation with a balanced salt solution (10 ml/kg/hour, Jonosteril^®^, Fresenius Kabi, Bad Homburg, Germany) via an internal jugular venous catheter inserted and tunnelled subcutaneously to the nape of the neck 24 hours before induction of sepsis.

### Clinical characterization of septic animals

To assess the severity of sepsis at the time point of investigation (15 hours post infection), the clinical status was evaluated as described in detail previously [14]. In addition, the volume of peritoneal fluid (ml) and fibrin film generation (scoring 0 to 3) were determined before surgical preparation for intravital microscopy. For whole blood analyses and plasma collection, heparin or ethylenediaminetetraacetic acid (EDTA) was used as the anticoagulant as warranted for the particular test. Blood samples were analyzed immediately after collection using an automated veterinary hematology analyzer (PocH-100iV Diff; Sysmex, Leipzig, Germany). For plasma collection, blood was centrifuged at 4,700 × g and 4°C for 10 minutes and the plasma immediately snap-frozen in liquid nitrogen and stored at -80°C for further analyses. Laboratory markers of organ dysfunction were analyzed using an automated clinical chemistry analyzer (Fuji Dri-Chem 3500i; Sysmex). IL-6 cytokine levels were determined using Rat-Quantikine enzyme-linked immunosorbent assay kits (R and D Systems, Wiesbaden, Germany) according to the manufacturer's instructions.

### Dyes for analysis of hepatocellular transport

Due to their exclusively hepatobiliary clearance as well as their fluorescent properties [12,15] ICG (Pulsion Medical Systems SE, Feldkirchen, Germany) and the benzopyrylium-based hemocyanine dye DY635 (Dyomics GmbH, Jena, Germany) were applied for assessment of hepatocellular transport by intravital microscopy. ICG was chosen due to its known pharmacokinetics [15] and its established use in the clinical setting [16]. ICG handling by the liver is predicted from its plasma disappearance curve and represents a widely used method for liver function testing. DY635 is also primarily hepatobiliary excreted and, therefore, suitable for analysis of hepatocellular transport with the advantage of better visualization of hepatobiliary transport due to its physicochemical properties [12]. However, it is not suitable for indirect visualization of hepatobiliary excretory function by transcutaneous whole body NIRF imaging due to the shorter wavelength of its fluorescence emission spectrum with concomitant lower tissue penetration.

Dyes were administered via the jugular vein catheter and studied at equimolar dosages (14 pmol/g body weight) which have been identified as optimal concentrations for intravital microscopy in preliminary studies (data not shown).

### Assessment of hepatocellular transport by direct detection of both dyes in bile

Fifteen hours post insult, animals were anesthetized and a laparotomy for cannulation of the choledochal duct with a polyethylene catheter was performed. For intermittent analysis of the dye concentration (*n *= 12 per dye, that is, *n *= 6 sham and *n *= 6 sepsis) in blood and bile after administration, samples were drawn at baseline and at 15 and 30 minutes. To assess dye concentrations a standard curve was generated in the respective fluid (plasma or bile) without the presence of the dye using a fluorophotometer (BMG Labtech GmbH, Offenburg, Germany). In addition, cumulative bile flow was calculated by weighing the collected bile.

### Assessment of hepatocellular transport by intravital fluorescence microscopy of the liver

In additional experiments, sinusoidal delivery and biliary clearance of both dyes (*n *= 8 per dye, that is, *n *= 4 sham and *n *= 4 sepsis) were visualized by using an inverted epifluorescence microscope (AxioObserver^® ^Z1; Zeiss, Jena, Germany) as described in detail previously [12]. In brief, 15 hours post insult, animals were anesthetized and a laparotomy was performed in order to exteriorize one liver lobule. Using an x40/0.6 objective lens, the liver surface was epi-illuminated with a 100-watt mercury lamp with two filter sets: 365- to 395-nm excitation and 445- to 450-nm emission band pass filters (4',6-diamidino-2-phenylindole (DAPI) filterset) were applied for visualization of the liver architecture (autofluorescence resulting from hepatocellular reduced pyridine nucleotides (NADH)). Depending on the injected dye, 775- to 805-nm excitation and 845- to 855-nm emission band pass filters (ICG) or 630- to 660-nm excitation and 650- to 690-nm emission band pass filters (DY635) were used.

Eight pericentral and eight periportal regions of each animal were assessed. Optimal gain, exposure time and enhancement settings were defined in preliminary studies. After acquisition of baseline images, the dye was administered and images were captured every five minutes over a 30-minute period. Fluorescence intensities of both dyes were quantified densitometrically and are expressed as the relative decrease of specific fluorescence intensity (%).

For evaluation of the hepatic microcirculation, five 20-second video sequences per animal were recorded with the x20 objective lens and the DAPI filterset. Sinusoidal perfusion failure was determined by evaluating the predominant flow classified as absent, sluggish, normal or brisk in sinusoids crossing a 100 μm circle by two independent investigators blinded to the experimental group.

### Assessment of hepatocellular transport by non-invasive near-infrared whole-body fluorescence (NIRF) imaging

Due to superior fluorescent properties for NIRF imaging, ICG alone could be used for transcutaneous assessment of hepatocellular transport. The whole-body NIRF imaging device (Maestro™, Cri, Woburn, MA, USA) was equipped with a 710- to 760-nm excitation band pass and an 800 nm emission long pass filter.

Animals (*n *= 5 sham and *n *= 5 sepsis) were fed with a specific manganese-reduced diet over a four-week period prior to commencing the experiments (to avoid interference by the near-infrared properties of manganese). These animals were treated and instrumented prior to experiments as described above. In addition, the abdominal region of all animals was shaved prior to the experiment. Fifteen hours post insult, ICG was administered intravenously (14 pmol/g body weight). Fluorescence emission was recorded before and at 2, 4, 6, 8, 10, 30, 90, 150 and 300 minutes after injection of ICG with the whole-body NIRF imaging system under short anesthesia. Subsequently, a laparotomy was performed and images were taken of the exposed abdominal cavity.

Fluorescence intensities at the upper right quadrant of the abdomen (liver) and at both lower quadrants of the abdomen (bowel) were semi quantitatively determined in manually selected circular regions of interest (ROIs) drafted with the same standard. Analysis was performed by an investigator blinded to the experimental groups. Fluorescence intensities are indicated as scaled counts/second, reflecting count levels after scaling for the effects of exposure time, binning and camera gain.

### Statistics

All data were examined for normal distribution (Kolmogorow-Smirnov test) and subsequent tests were applied as appropriate. Differences between groups were analysed by t-test or chi square test, as appropriate. For analysis of time-dependent changes within groups, the Friedman test was applied. Data are presented as mean ± standard error of the mean (SEM) or percentages, respectively. A *P *value < 0.05 was considered significant.

## Results

### Polymicrobial sepsis resulted in liver injury, severe cholestasis and impaired microcirculation

Septic animals displayed clinical signs of disease including a substantial decrease in activity and accumulation of peritoneal exudate as well as laboratory signs of systemic inflammation paralleled by multiple organ dysfunction (Table 1), including hepatocellular injury, increased markers of cholestasis and a reduced cumulative bile flow (Table 2).

**Table 1:** Assessment of disease severity and laboratory markers of sepsis-associated inflammation and organ injury and dysfunction

		sham	sepsis
		
	Unit	mean ± SEM	mean ± SEM
Activity score	1 (active) to 5 (lethargic)	1.0 ± 0.0	3.9 ± 0.2^a^
Peritoneal exudate	ml	0	5.3 ± 0.5^a^
Fibrin generation	0 (none) to 3 (massive)	0	2.6 ± 0.2^a^
White cell count	× 10^3^/μl	5 ± 0.4	0.9 ± 0.1^a^
Platelets	× 10^3^/μl	504.4 ± 32.1	294.7 ± 53.1^a^
IL6	pg/ml	407 ± 88	6,013 ± 2426^a^
LDH	IU/l	233.3 ± 25.7	695.4 ± 65.8^a^
Ammonia	μmol/l	200.2 ± 21.4	278.4 ± 35.9
AST	IU/l	76.3 ± 11.7	202.3 ± 43.5^a^
Albumin	g/dL	2.6 ± 0.1	1.5 ± 0.1^a^
Hematocrit	%	40.2 ± 0.6	49.4 ± 1.2^a^

^a^*P *< 0.05 compared to sham. AST, aspartate aminotransferase; IL6, interleukin-6; LDH, lactate dehydrogenase; SEM, standard error of the mean. *N *= 12 per group.

**Table 2:** Laboratory markers of liver injury and sepsis--associated cholestasis

		sham	sepsis
		
	Unit	mean ± SEM	mean ± SEM
Cumulative bile flow	μl·kg^-1^·min^-1^	45.0 ± 5.0	20.3 ± 3.5^a^
GGT	IU/l	3.4 ± 0.6	13.2 ± 2.1^a^
ALT	IU/l	32 ± 5.2	90.6 ± 16.8^a^
Total bilirubin	μmol/l	7.1 ± 0.6	15.2 ± 1.3^a^

^a^*P *< 0.05 compared to sham. ALT, alanine aminotransferase; GGT, gamma-glutamyl-transferase; SEM, standard error of the mean. *N *= 12 per group.

**Figure 2:**
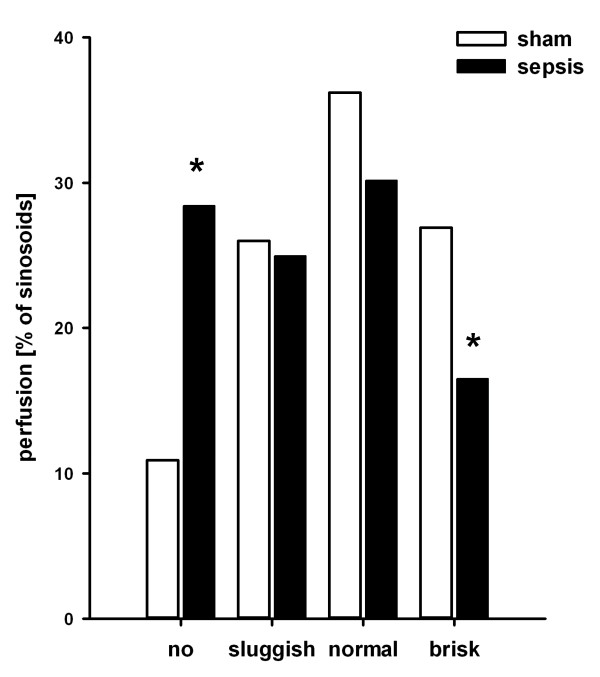
**Effects of polymicrobial sepsis on sinusoidal microcirculation**. Sinusoidal microcirculation (*n *= 4 animals/group, 5 regions of interest/rat) was assessed by intravital microscopy 15 hours after induction of sepsis. Bars represent the proportion of non-perfused sinusoids or velocity of sinusoidal prefusion, respectively. Open bars indicate sham-operated controls; filled bars indicate septic animals. *P < 0.05 for sham versus sepsis ('no': *P *= 0.009; 'brisk': *P *= 0.02). Septic animals had an overall shift to reduced sinusoidal perfusion.

Polymicrobial sepsis resulted in a substantial impairment of sinusoidal perfusion with a profound increase in the number of non-perfused sinusoids and a decreased portion of normal or brisk perfused sinusoids (Figure 2).

### Sepsis-associated impairment of hepatobiliary excretion is reflected in low recovery rates of DY635 and ICG in bile

**Figure 3:**
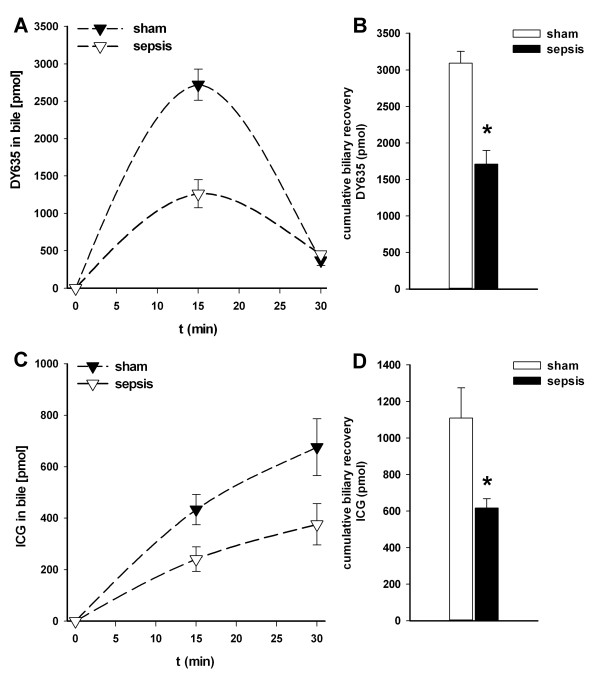
**Effects of polymicrobial sepsis on biliary excretion of two xenobiotics (DY635, ICG)**. Kinetics of (**A**) DY635 and (**C**) ICG with respect to biliary excretion in sham-operated as opposed to septic rats over an observation period of 30 minutes after intravenous administration (14 pmol/g; *n *= 6 animals/group; 15 hours after induction of sepsis). Cumulative biliary excretion of (**B**) DY635 and (**D**) ICG, respectively. *P < 0.05 for sham versus sepsis (DY635: *P *≤ 0.001; ICG: *P *= 0.013). In septic animals biliary recovery of the two dyes was significantly reduced. ICG, indocyanine green.

Peak plasma levels of both dyes one minute after administration were not different between sham and septic animals (DY635 sham versus sepsis: 253 ± 13 versus 325 ± 38 nM, non-significant (n.s.); ICG sham versus sepsis: 129 ± 11 versus 85 ± 12 μM, n.s.), and 15 minutes after administration both dyes were no longer detectable in plasma. However, excretion of both dyes differed. DY635 and ICG appeared more rapidly and at higher concentrations in the bile of sham animals (Figure 3A, 2C). Cumulative biliary recovery of both dyes over the observation period of 30 minutes showed a significantly higher absolute amount of substance recovered in sham compared to septic animals (DY 635 sham versus sepsis (Figure 3B): 3,092 ± 161 versus 1,711 ± 186 pmol, P ≤ 0.001; ICG sham versus sepsis (Figure 3D): 1,109 ± 167 versus 616 ± 51 pmol, *P *= 0.013). Consequently, 59% and 31% (DY635) as well as 20% and 11% (ICG) of the administered amount of substance could be recovered within 30 minutes in sham and septic animals, respectively.

### DY635 and ICG are rapidly cleared from plasma with concomitant accumulation in hepatocytes under conditions of sepsis

**Figure 4:**
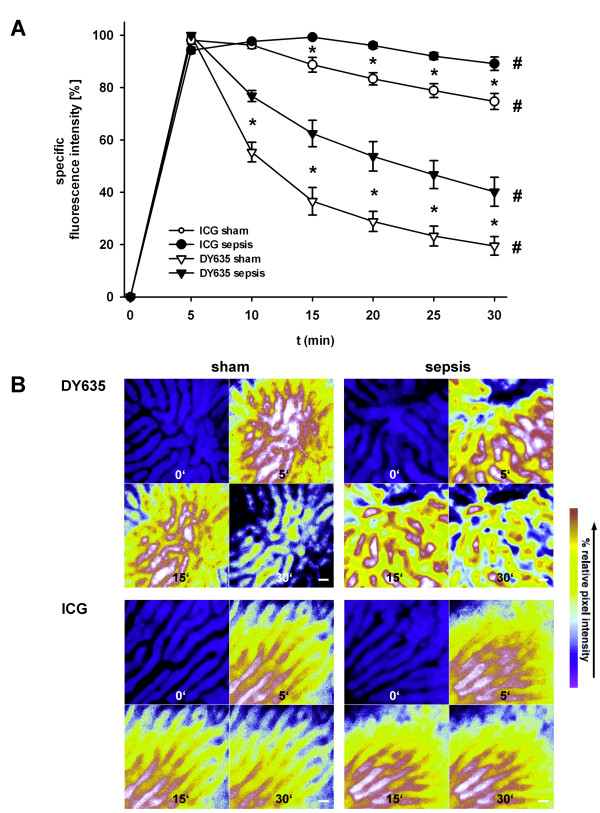
**Direct visualization of sinusoidal perfusion, hepatocellular uptake and biliary excretion of DY635 and ICG in polymicrobial sepsis assessed by intravital fluorescence microscopy**. (**A**) Kinetics of uptake and excretion of equimolar amounts of both dyes (14 pmol/g) by the liver as measured by densitometric assessment of the dyes (*n *= 4 animals/group, 8 periportal and 8 pericentral regions of interest/rat; 15 hours after induction of sepsis). Individual grey density maxima were set to 100. Depicted data represent precentage values of individual maximum. *P < 0.05 for sham versus sepsis. #*P *< 0.05 for within-group comparison. (**B**) Representative images (original magnification x400) of the same field of view of a liver lobule at baseline and at 5, 15 and 30 minutes after administration of DY635 (upper panel) and ICG (lower panel), respectively. Bar indicates 20 μm. For ICG, 775- to 805-nm excitation and 845- to 855-nm emission band pass filters and for DY635 630- to 640-nm excitation and 650- to 690 emission band pass filters were used. Images were pseudocolored to reveal differences in pixel intensity. Pseudocolored white-red (green-blue) regions reflect areas with high (low) grey densities. For the first image, 365- to 395-nm excitation and 445- to 450-nm emission band pass filters were applied for visualization of the liver architecture (pseudocolor blue). Black areas represent the sinusoids whereas blue areas represent hepatocytes. Hepatocellular uptake of the dyes occurs rapidly after intravenous administration. Then dye concentrations decrease over time reflecting hepatobiliary excretion. In comparison to sham animals, hepatobiliary excretion is impaired in septic animals reflected by the slower decrease of fluorescence intensity. While we could not detect a different uptake of DY635 between septic and sham animals, uptake of ICG seems to be slower as peak fluorescence in septic animals was postponed from 5 to 15 minutes. ICG, indocyanine green.

Assessment of hepatocellular transport by intravital microscopy revealed no differences in the uptake of DY635 from blood between sham and septic animals (Figure 4A, ascending part of the graph): five minutes after administration, DY635 reached maximum concentrations in the liver. Specific fluorescence of DY635 decreased to 20 ± 3.6% in sham and 40 ± 5.5% in septic animals within 30 minutes (*P *= 0.02). ICG exhibited prolonged uptake times in septic (peak at 15 minutes) compared to sham-operated animals (peak at 5 minutes) (Figure 4A, ascending part of the graph). Canalicular excretion of ICG was significantly delayed in septic rats. The relative decrease of specific fluorescence of ICG at 30 minutes equaled 75 ± 3.1% and 89 ± 2.6% (*P *= 0.011) in sham and septic rats, respectively. The kinetics of both dyes could reliably differentiate septic animals from sham-treated controls. Thus, biliary clearance for both dyes was impaired under conditions of sepsis and resulted in an accumulation of both dyes in hepatocytes (Figure 4B, Additional file 1).

### Hepatocellular transport is specifically impaired at the canalicular pole

**Figure 5:**
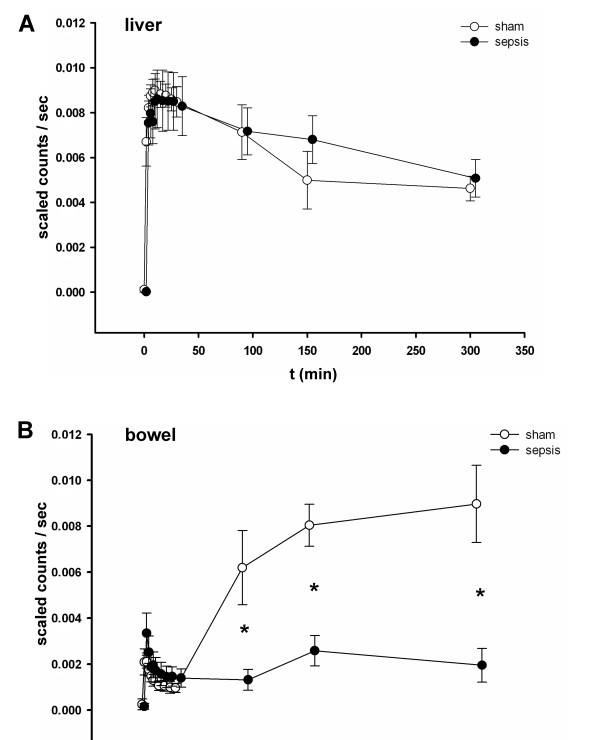
**Transcutaneous whole-body near-infrared fluorescence (NIRF) imaging of ICG enables non-invasive assessment of impaired hepatobiliary excretion in polymicrobial sepsis**. Fifteen hours post induction of sepsis, ICG fluorescence was assessed transcutaneously by a whole body NIRF imaging device over a time period of 300 minutes (semi-quantitative analysis, *n *= 5 animals/group) at the indicated timepoints. *P < 0.05 for sham versus sepsis (90.min: *P *= 0.019; 150.min: *P *= 0.001; 300.min: *P *= 0.005). Kinetics of emitted fluorescence intensities of ICG detected in (**A**) the liver region or (**B**) the bowel region of the abdomen. While different fluorescence intensities between sham and septic animals could not be demonstrated in the liver region, fluorescence intensities in the bowel region significantly differed between sham and septic animals. This hints towards a substantial impairment of biliary ICG excretion in septic animals. ICG, indocyanine green.

**Figure 6:**
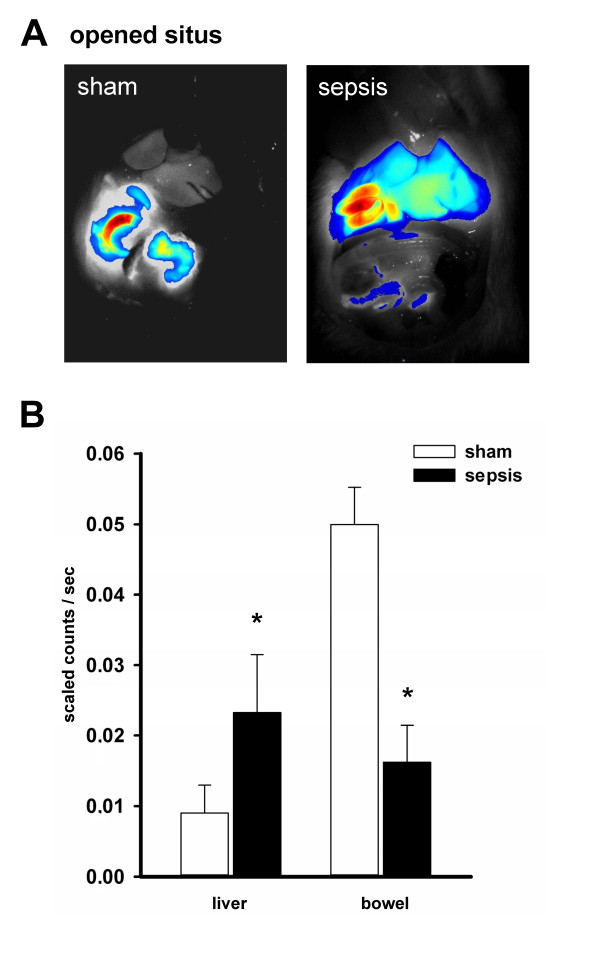
**Whole-body NIRF imaging at the opened *situs *clearly demonstrates an impairment specifically at the canalicular pole under conditions of sepsis**. (**A**) Depicted are representative images taken 300 minutes after the experiment at the opened *situs *confirming an accumulation of ICG in the liver of septic animals, with an almost absent fluorescence signal in the bowel. The overlay is of fluorescence (false colors: blue (low fluorescence intensity) and red (high fluorescence intensity)) and white-light images. (**B**) Depicts the detected fluorescence intensities in liver and bowel of all animals at the open *situs *300 minutes after administration of ICG. *P < 0.05 for sham versus sepsis ('liver': *P *= 0.008; 'bowel': *P *= 0.002).

Next, hepatocellular transport of ICG was non-invasively evaluated by whole-body NIRF imaging over the prolonged observation period of five hours in sham and septic animals. While transcutaneous assessment of ICG fluorescence in the liver region of the abdomen failed to reveal any differences between sham and septic rats (Figure 5A), a significant increase of ICG fluorescence from the 30th minute on could be detected in the bowel region of the abdomen in sham but not in septic animals (Figure 5B), confirming a sepsis-associated failure of canalicular excretion. In contrast to the transcutaneous approach, biooptical NIRF recordings in all animals with an opened abdominal *situs *at five hours post administration of the dye (Figure 6A) confirmed an accumulation of ICG in the liver (Figure 6B) and underlined the significantly lower ICG fluorescence in the bowel region of the abdomen in septic animals.

## Discussion

In the present study, we applied *in vivo *imaging in combination with fluorophotometric determination of ICG and DY635 kinetics, including uptake, intracellular transport and excretion, enabling us to analyze the efficiency of the hepatocellular transport systems under conditions of sepsis. Visualization of the hepatobiliary excretory function in septic animals demonstrated a profound impairment of hepatocellular transport, specifically at the canalicular pole.

Our findings of a sepsis-associated impairment of hepatic excretory function specifically at the canalicular pole support and strengthen recent reports about mechanisms of a disturbance of the hepatocellular transport machinery in early sepsis. As indicated above, an efficient hepatobiliary excretion is the net result of many functioning variables. Accordingly, the delayed dye clearance in septic animals observed in this study may be due to compromised distribution or altered uptake kinetics. However, uptake of both dyes was not severely impaired despite a significantly impaired microcirculation in septic animals. Albeit slightly prolonged uptake times could be detected for ICG, both dyes were completely cleared from plasma within 15 minutes. Moreover, no renal clearance of the potentially unbound fraction of either DY635 or ICG (data not shown) was observed. Therefore, the effect of a compromised microcirculation seems to be of limited significance under the given conditions, albeit this might be different in animals with an even more profoundly impaired microcirculation, that is, in septic shock with severe hypotension or in chronic liver disease with increased intrahepatic vascular resistance and consecutive portal hypertension leading to porto-systemic shunting.

Analysis of cumulative recovered bile at 30 minutes revealed significantly lower amounts of both dyes in septic animals strongly suggesting an impaired transport at the canalicular pole. Applying non-invasive whole-body NIRF imaging enabled us to indirectly assess the canalicular pole by measuring emitted fluorescence from the bowel region. In septic animals, the impairment of excretion at the canalicular pole became obvious since almost no fluorescence could be detected in this region five hours after administration.

Intravital microscopy of the liver revealed comparable distribution and uptake phases for both dyes in sham and septic animals, but a markedly different hepatobiliary excretion of DY635 compared to ICG. Moreover, the appearance and cumulative recovery of DY635 in bile also differed from ICG with a more rapid appearance and a quantitatively higher amount recovered in both sham and septic animals. Whether accelerated transmembrane transport or intracellular binding and transport that differ from ICG are responsible for the much faster hepatobiliary clearance of DY635 remains speculative. Nevertheless, uptake of both dyes within 15 minutes could be confirmed as well as a profound hepatic excretory dysfunction resulting in hepatic accumulation of both dyes in septic as opposed to sham animals.

In contrast to DY635, the mechanisms of ICG transport are well defined. ICG uptake in the rat is mediated by a mechanism mainly involving the organic anion transporting polypeptide-1 (Oatp1) [17,18]. After intracellular binding to ligandin(s) ICG is transported to the canalicular surface of the hepatocyte and is mainly excreted via Mdr2 [19]. Rodent Mdr2 and its human homolog MDR3 translocate phospholipids and several xenobiotics from the cytosolic to the luminal leaflet of the canalicular membrane [20].

A recent study by our group revealed a differential gene expression of basolateral and canalicular transporters for ICG and bilirubin in human precision-cut liver slices [4]: whereas genes encoding prototypical canalicular transporters (MRP2, MDR3) were down-regulated after cytokine stimulation and glutathione depletion, genes encoding the basolateral transporters (OATP1, OATP2) were maintained or even up-regulated, suggesting maintained uptake of ICG from sinusoidal blood but impaired ability to transport the bilirubin/ICG at the canalicular pole. In addition, in a rodent model of sepsis not only a sepsis-associated down-regulation of canalicular transporters, such as Mrp2 (affecting transcription, translation and post-translational modification), but also an association of the degree of down-regulation with predicted outcome was demonstrated. In particular, posttranslational changes, that is, a withdrawal of microvilli and related Mrp2 transporters from the canalicular membrane, could be detected under septic conditions [7]. The differential susceptibility of the two polar surfaces of hepatocytes is further supported by findings in liver transplant recipients: in those patients with remote organ failure and/or primary graft dysfunction, biliary ICG excretion dropped dramatically, whereas ICG uptake rate was far less reduced [4]. Altogether, these findings can provide reasonable mechanistic explanations for the observed impairment of hepatobiliary excretion in this study. However, whether the impaired canalicular ICG excretion observed results solely from down-regulation of Mdr2 or if there is an additional functional impairment remains uncertain. Also a disturbed energy state with ATP-depletion due to the detected perfusion disorders leading to malfunction of ATP-dependent canalicular transporters is possible.

Measurement of the plasma disappearance rate of ICG (PDR_ICG_), a quantitative liver function test, renders a complex measure of both sinusoidal perfusion as well as basolateral hepatic (cell) membrane function. Thus, it reflects the functional reserve of hepatocytes which have access to perfusion. While measuring PDR_ICG _represents a widely used and approved method for liver function testing in acute and chronic hepatic disorders, the test lacks detailed information about liver-bile interactions, thereby failing to evaluate specifically canalicular transport, an increasingly recognized component of sepsis-associated excretory dysfunction [3,4,7,10,16]. In the present study, we have successfully applied a non-invasive biophotonic technique using ICG for assessment of the canalicular pole which could not be monitored in the routine clinical setting up to now. Limitations remain, which impede an immediate transfer of this optical method into clinical practice as current NIRF imaging systems lack an adequate anatomical resolution and tissue penetration of fluorescence is restricted. Although a prognostic value of PDR_ICG _in critically ill patients, even in the early course of the disease, has been demonstrated [4,21,22], the test is, according to our results and in line with findings in a porcine model of endotoxemia [3] as well as in liver transplant recipients [4], likely to underestimate true hepatobiliary excretory dysfunction.

While it cannot be presumed that the transport of relevant xenobiotics is affected in the same way as the dyes used in this study, our present data may have major implications for the understanding of drug-induced liver injury, especially in the critically ill. Little information is available concerning the altered pharmacokinetics of drugs routinely administered in the ICU; concerns about their safety and toxicity are increasing. For example, oral single-dose administration of atorvastatin in septic patients resulted in supratherapeutic plasma concentrations for up to 24 hours [23]. Transmembrane transporters are important determinants for hepatic drug clearance that are mainly responsible for the alterations described herein. Specifically, an impairment of transporters located at the canalicular pole may lead to intracellular accumulation of hepatotoxic compounds in septic conditions potentially contributing to drug-induced liver injury in the critical ill.

## Conclusions

Taken together, our data confirm and extend the differential susceptibility of the two polar surfaces of hepatocytes. The liver's capacity to transport and specifically excrete organic anions seems to represent a particularly vulnerable process in septic rodents and presumably in critically ill patients with sepsis, which should be considered in 'critical care pharmacology.' In addition, monitoring of the canalicular pole might serve as an early warning signal of hepatocellular dysfunction.

## Key messages

• Hepatocellular transport is impaired in sepsis due to an alteration of the hepatic transport machinery.

• The two polar surfaces of hepatocytes exhibit a differential susceptibility with the canalicular pole being more vulnerable under conditions of sepsis.

• Critical care pharmacology and diagnostics should consider the specific impairment of canalicular excretion since this may lead to a hepatic accumulation of potentially hepatotoxic compounds.

## Abbreviations

DAPI: 4',6-diamidino-2-phenylindole; ICG: indocyanine green; IL: interleukin; Mrp: multidrug resistance-associated protein; Mdr: multidrug resistance protein; NADH: nicotinamide adenine dinucleotide; NIFR: near-infrared fluorescence; Oatp: organic anion transporting polypeptide; PDR: plasma disappearance rate; SEM: standard error of the mean.

## Competing interests

MB is a member of the Medical Advisory Board of Pulsion Medical Systems SE, a manufacturer of ICG. AK received a study grant from Pulsion Medical Systems SE. MB, FAG and PR hold a patent for application of polymethine fluorescent dyes (including DY635) for monitoring organ dysfunction. The invention further relates to a kit for determining an organ function with a marker dye.

## Authors' contributions

FAG and PR participated in the experimental design, performed animal studies, analyzed and interpreted the data and drafted the manuscript. IH and RAC participated in the experimental design, gave technical support, interpreted the data and revised the manuscript. MB and AK designed the study concept, supervised the research, interpreted the data and reviewed the manuscript for important intellectual content. All authors read and approved the final version of the manuscript.

## Supplementary Material

Additional file 1 **Visualization of hepatocellular transport by intravital fluorescence microscopy**. Sinusoidal delivery and biliary clearance of DY635, a fluorescent dye that is primarily hepatobiliary excreted, in a representative sham and septic animal 15 hours post insult. After intravenous administration of the dye, images of the same region of the liver were captured every five minutes (x40/0.6 objective lens; the liver surface was epi-illuminated with a 100-watt mercury lamp with two filter sets: i) 365- to 395-nm excitation and 445- to 450-nm emission band pass filters (DAPI filterset) were applied for visualization of the liver architecture (autofluorescence resulting from hepatocellular reduced pyridine nucleotides (NADH)); ii) 630- to 660-nm excitation and 650- to 690-nm emission band pass filters (CY5 filterset) were applied for visualization of DY635). In the sham animal, DY635 displayed a rapid uptake with immediate excretion into the bile canaliculi while excretion was impaired under conditions of sepsis. Click here for file
